# Supporting Physical and Mental Health in Rural Veterans Living With Heart Failure: Protocol for a Nurse-Led Telephone Intervention Study

**DOI:** 10.2196/63498

**Published:** 2025-03-26

**Authors:** Lucinda J Graven, Laurie Abbott, Josef V Hodgkins, Thomas Ledermann, M Bryant Howren

**Affiliations:** 1 College of Nursing Florida State University Tallahassee, FL United States; 2 College of Education, Health, and Human Sciences Florida State University Tallahassee, FL United States; 3 Carver College of Medicine University of Iowa Iowa City, IA United States; 4 Iowa City VA Health Care System Iowa City United States

**Keywords:** heart failure, veterans, problem-solving, self-care, heart failure symptoms, depression, anxiety, HRQOL, health-related quality of life, stress, resilience, coping, mental health, nurse-led intervention, social support, telehealth, chronic disease management

## Abstract

**Background:**

Heart failure (HF) remains a disease of notable disparity for rural veterans, despite recent advancements in clinical treatment. Managing HF in the home is stressful and complex for rural veterans who experience unique barriers to optimal physical and mental health, necessitating adequate support and problem-solving skills.

**Objective:**

This study aims to (1) adapt, to the rural sociocultural context, a culturally sensitive, tailored, telephone support and problem-solving intervention (CARE-HF [Supporting Physical and Mental Health in Rural Veterans With Heart Failure]) using findings from preliminary qualitative research and (2) evaluate the effects of CARE-HF on problem-solving and physical and mental health outcomes among rural veterans with HF.

**Methods:**

This study involves a repeated-measures, single-group design. The intervention content was adapted and tailored to the rural sociocultural context using preliminary qualitative data and guided by the Theories of Social Problem-Solving and Stress, Appraisal, and Coping. Veterans are recruited from Veterans Administration home-based cardiac rehabilitation clinics, cardiology clinics that serve veterans, veterans-based community resource centers, and social media campaigns. Veterans with HF (N=100) receive the CARE-HF intervention. This nurse-led intervention comprises 8 telephone sessions that use a five-step, problem-solving process to manage common HF problems in the home: (1) identifying the problem and viewing it in a positive manner, (2) goal setting, (3) generating potential strategies for problem management, (4) choosing and implementing strategies to manage the problem, and (5) evaluating strategy effectiveness. Veterans receive initial problem-solving training during the first session, with follow-up sessions focusing on problem-solving skill reinforcement and assisting veterans in applying these principles to manage self-identified, HF-related problems experienced in the home. Data are collected at baseline and 3, 6, 12, and 18 months from baseline on problem-solving and outcomes of interest (ie, HF self-care; HF symptoms; health care utilization; depressive symptoms; anxiety; HF-specific, health-related quality of life; stress; resilience; and coping). Demographic data will be analyzed using descriptive statistics and multilevel growth curve modeling with restricted maximum likelihood estimation to compare a series of models using Akaike information criteria and Bayesian information criteria fit indices while controlling for covariates.

**Results:**

Recruitment started in April 2023. As of December 2024, we have enrolled 56 veterans. Recruitment is anticipated to end in June 2025, with data collection continuing until all enrolled veterans have completed the 18-month follow-up period.

**Conclusions:**

Adapting and testing a culturally sensitive, tailored, telephone intervention to aid support and problem-solving in the home has the potential to provide individualized care to rural veterans where they reside, thereby reducing travel burden while also increasing access to evidence-based care programs. If effective, telephone support and problem-solving interventions could be a low-cost, accessible method to improve physical and mental health in rural veterans with HF.

**Trial Registration:**

ClinicalTrials.gov NCT05839067; https://clinicaltrials.gov/study/NCT05839067

**International Registered Report Identifier (IRRID):**

DERR1-10.2196/63498

## Introduction

### Background

Heart failure (HF) is a chronic, progressive disease of notable disparity for rural veterans despite recent advancements in clinical treatments [1] and overall improvements in HF-related outcomes [2]. Incidence, hospitalization, and mortality rates continue to be highest in rural versus urban individuals with HF [2,3]. In rural veterans, specifically, HF is the second leading cause of cardiovascular-related hospitalization, accounting for about 30% of total hospitalizations [4]. Lower levels of health literacy, higher rates of food insecurity, the presence of food deserts, the lack of community support services, poverty, and limited health care access contribute to the disparities in HF-related outcomes in rural veterans [5-7] and influence physical and mental health [7].

Rurality impacts patients’ ability to maintain optimal HF self-care and disease management in the home. Specifically, rural veterans with HF experience unique challenges, which are stressful, such as difficulties in obtaining fresh fruits and vegetables and low-sodium foods, which are a vital component of the HF dietary regimen [8]. The lack of local health care services and pharmacies, as well as public transportation, adds to the complexity of managing HF in a rural area [8] and increases the travel burden for veterans associated with receiving medical care [9]. In fact, only 9% of physicians practice in rural areas, which is significant given that 20% of the population in the United States lives in rural areas [6]. These rural-related social determinants of health play a crucial role in the ability to maintain optimal HF self-management and influence patient outcomes in rural veterans with HF [7].

Likewise, depression and anxiety are influenced by the progression of HF [10], with increased symptoms and decreased physical functioning contributing to poor mental health [11,12], and are associated with higher mortality and hospitalization rates [10,13]. Notably, depression and suicide rates are highest in rural patients compared to their urban counterparts, yet only 10% of mental health professionals practice in rural areas, leaving many rural veterans without access to mental health care [14] and placing them at even greater risk for poor HF outcomes [10,13]. Thus, rural veterans living with HF lack substantial support in aiding disease management and mitigating the negative emotions that often accompany living with HF, necessitating the development of novel interventions to support physical and mental health in the home.

Social support and problem-solving are essential coping resources that aid stress management, support resiliency, and enhance coping processes [15,16]. In rural veterans with HF, social support and problem-solving skills are critical to maintaining physical and mental health [8,17,18]. Patients with HF need to appraise and manage diverse symptomatology, adhere to dietary restrictions, cope with negative emotions, and negotiate daily activities [11,12]. Rural veterans with HF also need to successfully address rural-related challenges and barriers that undermine overall health [7]. Previous research suggests that the use of rational, systematic problem-solving strategies to manage HF-related problems is associated with better HF self-care [8,17] and may reduce HF symptom severity [18] and mental distress [17]. However, rural veterans often lack access to support and problem-solving interventions in their communities. Furthermore, when such services are available, they typically are not tailored to the rural sociocultural and educational context, nor are they able to address the specific emotional needs of veterans [19].

Culturally sensitive, tailored interventions that provide support and focus on enhancing problem-solving skills [16] hold considerable promise for increasing uptake in rural veterans with HF. Tailored support and problem-solving programs may be beneficial for veterans who need more assistance outside of a “standardized, one-size-fits-all” disease self-management program. Furthermore, the use of telephones allows for an easy-to-access, cost-effective, and successful modality for delivering such interventional programs to rural areas where access to broadband internet may still be problematic [20-22].

Our previous research assessed the preliminary efficacy of a 12-week, nurse-led, tailored, telephone-based, problem-solving and support intervention (Coping in Heart Failure [COPE-HF] Partnership) for patients with HF on changes in self-care, HF symptoms, depressive symptoms, and health care utilization in a three-arm randomized clinical trial [17,18]. Participants in the COPE-HF Partnership were all recently discharged from an acute care facility for issues related to HF and resided primarily in urban areas. A trained nurse interventionist partnered with the patient to manage HF-related self-care and disease management problems encountered in the home. Data were collected at baseline and 5, 9, and 13 weeks. The intervention was associated with improvements in self-care management; notably, patients had less health care utilization over the intervention period versus the other groups [17]. Patients who received the intervention also reported fewer HF symptoms, with a significant decrease in HF symptom severity over the intervention period [18]. Although not significant, improvements in depression were also found in the intervention group [17].

To prepare for the sociocultural adaptation of the CARE-HF (Supporting Physical and Mental Health in Rural Veterans With Heart Failure) intervention to the rural population, we conducted a qualitative study with rural patient–care partner dyads living with HF (n=11) to identify and describe the problems experienced in the home related to HF and associated management strategies [8]. The findings of this study showed that rural residents with HF experience significant problems related to self-care and disease management adherence due to the rural environment and lack of resources, as well as differences in cultural and personal values and beliefs related to health care. These problems resulted in a variety of physical and mental health sequelae and strained interpersonal relationships. However, most dyads developed effective workarounds and management strategies to overcome these challenges [8]. The problems and management strategies identified in this study provide the basis for the adaptation of intervention content for the rural population included in the CARE-HF intervention.

The CARE-HF also builds upon the COPE-HF Partnership intervention, in that it includes self-care and disease management components [17,18]. However, it differs in that the CARE-HF intervention is more holistically focused, also incorporating mental health, interpersonal, and social components. In addition, all components of the CARE-HF intervention are adapted to the rural sociocultural context and include specific problems and management strategies pertaining to physical and mental health drawn from the rural setting [8]. Because of the expanded focus of the CARE-HF intervention, additional study outcomes, not examined in the parent intervention, are included in the CARE-HF study.

### Conceptual Framework

**Figure 1:**
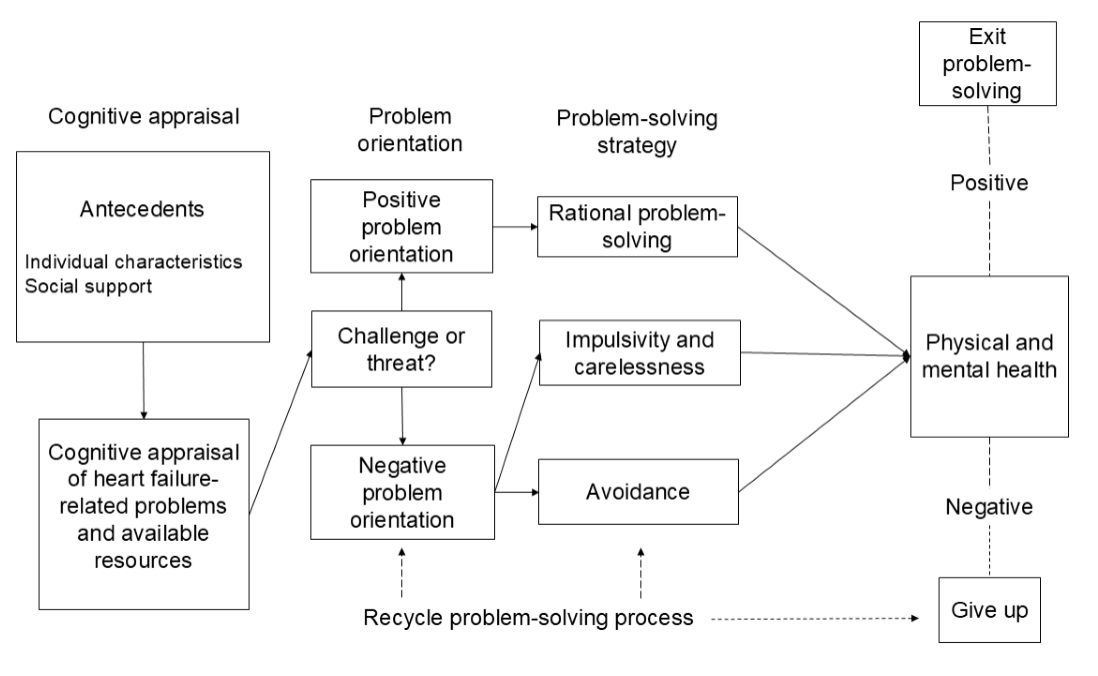
Conceptual model based on the Theories of Social Problem-Solving and Stress, Appraisal, and Coping.

The Theories of Social Problem-Solving [16] and Stress, Appraisal, and Coping [15] provide the foundation for the CARE-HF intervention (Figure 1). Rural veterans with HF encounter a unique variety of problems that require cognitive appraisal and appropriate problem-solving to successfully cope with HF-related challenges. The two-fold cognitive appraisal process is influenced by individual characteristics and consists of primary appraisal (determination of the significance of the problem) and secondary appraisal (evaluation of available resources) [15]. Effective problem-solving requires a positive problem-orientation approach which elicits rational problem-solving versus avoidance, impulsivity, and carelessness. Problem-solving involves accurate problem identification, generation of appropriate strategies, active decision-making, and strategy implementation and evaluation [16]. The goal of the CARE-HF intervention is to move rural veterans with HF toward a positive problem orientation and use of rational problem-solving strategies, thereby supporting a more adaptive problem-solving style and enhancing physical and mental health [15,16].

### Purpose, Aims, and Hypotheses

The overall purpose of this study is to adapt to the rural sociocultural context and test the efficacy of a tailored, telephone-based intervention (CARE-HF) to support the physical and mental health of rural veterans by enhancing disease self-management and coping processes. To accomplish this goal, our research aims are to:

1. Adapt the CARE-HF intervention content to the rural sociocultural context using findings from preliminary qualitative research.
2. Examine the effectiveness of the CARE-HF intervention on study outcomes (HF self-care, HF symptoms, health care utilization, depressive symptoms, anxiety, health-related quality of life, stress, resilience, and coping), process (problem-solving), and antecedent (social support) in a sample of rural veterans with HF (N=100) at baseline and 3, 6, 12, and 18 months from baseline.

Our hypotheses are as follows:

- Hypothesis 2a: Increases in HF self-care, health-related quality of life, resilience, coping, and problem-solving will be noted at 3 months from baseline and sustained throughout the 18-month follow-up period.
- Hypothesis 2b: Decreases in HF symptoms (frequency, severity, and interference with physical activity and enjoyment of life), health care utilization, depressive symptoms, anxiety, and stress will be seen at 3 months from baseline and sustained throughout the 18-month follow-up period.

## Methods

### Cultural Adaptation

To address aim 1, before recruitment and study initiation, steps were taken to adapt the CARE-HF intervention content to the rural sociocultural context using some aspects of the ADAPT-ITT (Assessment, Decision, Adaptation, Production, Topical Experts, Integration, Training, and Testing) model [23]. Specifically, qualitative findings from preliminary research in rural patients with HF [8,12] guided the context of problems and potential strategies included in the program materials and telephone intervention sessions. The problems presented during the card sorting task, a component of the sessions, are drawn directly from this preliminary rural research [8,12]. In addition, during the telehealth sessions, veterans’ values and beliefs are assessed and inform the potential strategies identified in the problem-solving process to enhance adherence and tailor the program to the unique needs of each rural veteran.

### Ethical Considerations

This study was approved by the Florida State University Institutional Review Board (protocol STUDY00003764) to collect verbal consent via the telephone according to the principles of the Declaration of Helsinki, with potential participants maintaining the opportunity to opt out of the study. Telephone sessions are recorded to monitor intervention fidelity. However, all participant data are deidentified and stored in accordance with the General Data Protection Regulation Rules to maintain privacy and confidentiality. Veterans enrolled in the study receive compensation in the form of US $20 gift cards to a superstore following each completed data collection point, for up to a total of US $100 in gift cards if the entire study is completed. No identifiable information or images of research participants will be included in research reports. The CARE-HF protocol is registered at ClinicalTrials.gov (NCT05839067).

### Design and Sample

This study includes a repeated-measures, single-group design. Veterans are recruited from Veterans Administration home-based cardiac rehabilitation clinics, cardiology clinics that serve veterans, veterans-based community resource centers, and social media campaigns using study flyers. Veterans with HF (N=100) will be recruited over 3 years, with the anticipated enrollment of 5 or more veterans per month. The desired sample size was based on a power analysis for repeated-measures ANOVA with 5 time points, α=.05, a medium effect size (f=0.25), and 80% power, plus oversampling for potential attrition (20%) estimated from studies involving patients with HF [17,18]. Veterans are eligible for participation if they (1) have a diagnosis of HF; (2) are aged 18 years or older; (3) have New York Heart Association functional class II-IV HF; and (4) can read, write, and communicate verbally in English, and excluded if they have a history of cognitive impairment.

### Data Collection

Telephone data collection is conducted by a trained research assistant who verbally asks each item and records participants’ responses in an online HIPAA (Health Insurance Portability and Accountability Act)–compliant Qualtrics database. Data are collected at baseline and 3, 6, 12, and 18 months from baseline. In addition to a sociodemographic and clinical survey (age, gender, comorbidities, education, and HF class), a set of self-report surveys is used to measure the study variables (Table 1). We also collect data on standard Veterans Administration Office of Rural Health metrics.

**Table 1:** Study variables.

Variables		Baseline	3 months	6 months	12 months	18 months
**Covariates**						
	Social support	✓				
**Process**						
	Problem-solving	✓	✓	✓	✓	✓
**Outcomes**						
	HF^a^ self-care	✓	✓	✓	✓	✓
	HF symptoms	✓	✓	✓	✓	✓
	Health care utilization		✓	✓	✓	✓
	Depressive symptoms	✓	✓	✓	✓	✓
	Anxiety	✓	✓	✓	✓	✓
	HF-specific, health-related quality of life	✓	✓	✓	✓	✓
	Stress	✓	✓	✓	✓	✓
	Resilience	✓	✓	✓	✓	✓
	Coping	✓	✓	✓	✓	✓

^a^HF: heart failure.

### Study Measures

#### Social Support

The 12-item Interpersonal Support and Evaluation List measures perceived belonging, tangible, and appraisal support. The 3 subscales will be combined to obtain a single index of perceived support, in addition to the evaluation of the individual subscales. Scores range from 0-36, with higher scores suggesting a higher perception of available support [24]. Previous studies support its construct validity using the original 40-item version [24] and its internal consistency reliability (α=.90) [25].

#### Problem-Solving

The 25-item Social Problem-Solving Inventory Revised-Short measures problem orientation and problem-solving style using 5 subscales: positive and negative problem orientation, rational problem-solving, impulsivity and carelessness, and avoidance styles. The 5 subscales will be combined to provide a total score, with higher scores representing more of an adaptive problem-solving style and lower scores suggesting more of a maladaptive style. Higher scores on the individual subscales suggest more of the problem-solving characteristics [26]. Research supports its validity [16,26] and its reliability (α=.91) [25].

#### HF Self-Care

The 39-item Self-Care of Heart Failure Index v 7.2 measures HF self-care across 4 subscales: self-care maintenance, symptom perception, symptom management, and self-efficacy (α=.73-.88 across all subscales) [27,28]. Scores are standardized (0-100), with higher scores on each subscale suggesting better self-care principles. Scores ≥70 on each subscale are considered adequate; improvement of ≥8 is considered clinically significant [27].

#### HF Symptoms

HF symptoms are measured using the Heart Failure Symptom Survey. This survey contains 14 common symptoms of HF, which are rated on an 11-point scale (0–10) across 4 domains (frequency, severity, interference with activity, and interference with quality of life) based on the last 7 days. Higher scores indicate more of the respective domain in relation to the specific symptom [29]. Empirical evidence supports its content validity [29] and reliability (α=.96) [25].

#### Health Care Utilization

Health care utilization is determined by the frequency of emergency department visits and readmissions for HF and assessed via self-report. There are acceptable levels of agreement between self-report and medical record data [30].

#### Depressive Symptoms

Depressive symptoms are measured using the 20-item Center for Epidemiological Studies—Depression Scale. Overall scores range from 0 to 60, with higher scores indicating the presence of more depressive symptoms. A cutoff score of 16 indicates an individual is at risk for some degree of depression. Previous studies support its validity (Radloff [31]) and reliability (α=.90) [25].

#### Anxiety

The Generalized Anxiety Disorder-7 scale is a valid and reliable (α=.92) 7-item scale that assesses the presence of anxious symptoms over the past 14 days, using a Likert scale to rate the frequency of the symptoms (0=not at all, 1=several days, 2=more than half of the days, and 3=nearly every day). Total scores range from 0 to 21, with higher scores indicating more severe anxiety symptomatology [32]. In this study, the following recommended anxiety severity categories will be used: none or normal (0-4), mild (5-9), moderate (10-14), and severe (15-21) [32].

#### Health-Related Quality of Life

The multidimensional 21-item Minnesota Living with Heart Failure Questionnaire measures the physical, socioeconomic, and emotional dimensions of HF-specific, health-related quality of life. This self-report survey asks respondents to evaluate the way they have felt in relation to the specific dimension over the last 4 weeks. Scores for the individual dimensions, as well as a total score, are obtained by summing the individual items. Higher scores suggest worse health-related quality of life relative to the specific dimension or overall. A change of ≥5 points is considered clinically meaningful [33]. Previous studies support its validity [33] and reliability (α>or=.80 across dimensions) [34].

#### Stress

The valid and reliable 10-item Perceived Stress Scale measures stress and includes questions relative to feelings and thoughts experienced during the last month using a Likert scale with scores ranging from 0 (never) to 4 (very often). Higher scores indicate higher levels of acute stress [35].

#### Resilience

The 25-item Five-by-Five Resilience Scale has 5 subscales that measure adaptability, emotion regulation, optimism, self-efficacy, and social support on a 5-point Likert scale (1=very inaccurate to 5=very accurate). Higher scores represent higher levels of resilience relative to the specific facet. Previous research shows adequate validity and reliability (α=.81-.93 across subscales) [36].

#### Coping

The Brief Coping Orientation to Problems Experienced is a 28-item scale that measures 14 coping strategies over 3 subscales (active emotional coping, avoidant emotional coping, and problem-focused coping) on a 4-point scale ranging from 1 (I haven’t been doing this at all) to 4 (I have been doing this a lot). Higher scores on the subscales suggest more use of the specific type of coping strategy [37]. Previous research has indicated the measure is valid [37] and reliable (α=.78) [38].

### Procedure and Intervention

#### CARE-HF

Veterans are trained to use social problem-solving skills to manage common HF-related problems in the home during 8 telephone sessions led by a trained registered nurse interventionist (NI).

#### Initial Telephone Session

Before the first session, veterans are mailed program materials and a manual presented at a sixth-grade reading level. To start the first telephone session, participants receive an overview of the program manual, tailored to the rural context, which includes information about social problem-solving principles. The manual also includes detailed real-life examples drawn from the rural setting of how to use the five-step, problem-solving process to evaluate and manage HF-related problems based on previous research (Table 2) [8]. Each example includes a positive problem-orientation approach and rational problem-solving strategies to address these HF-related problems. The NI uses fatigue as an example when discussing how to apply the iterative five-step, problem-solving process to manage actual HF-related problems. The five-step, problem-solving process is based upon the “Theory of Social Problem-Solving” and rational problem-solving principles and includes (1) identifying the problem and viewing it in a positive manner, (2) setting a goal related to problem management, (3) generating potential strategies to address the problem, (4) choosing and implementing one to two strategies to manage the problem, and (5) evaluating the effectiveness of chosen strategies [16].

Veterans are then asked to participate in a card-sorting task. Veterans are given a set of cards portraying HF-related problems identified in previous research (Table 2) [8,12] and asked to prioritize the cards from the highest priority problem to the lowest. Then, the NI will guide veterans in applying the problem-solving process to the HF-related problem identified as the highest priority. Next, the NI describes the benefits of reframing the problem in a positive manner and facilitates the selection of preliminary strategies to manage them. In doing so, the NI explores through discussion with the participants how their values and beliefs can be incorporated into potential strategies. Participants then choose one to two strategies on which to focus, the results of which are reviewed at the next session. The five-step, problem-solving process is reinforced throughout the intervention period in the follow-up telephone sessions.

**Table 2:** HF^a^-related problems and examples.

Problem categories	Examples
Monitoring and managing HF symptoms	Fatigue (tiredness) Forgetfulness Edema (swelling) Activity intolerance Difficulty breathing (shortness of breath)
Staying on your special diet program	Access to suitable foods Shopping for and cooking correct foods Limiting fluids (if recommended) Barriers to eating foods with less salt Personal food likes and dislikes Not sure how to read and understand food labels
Staying on your HF treatment program	Take daily weights Exercise regularly (as recommended) Keep regular health care visits Get immunizations (flu, RSV^b^, pneumonia, and COVID-19) Use oxygen (as directed) Stop smoking
Dealing with unhelpful emotions	Sadness Anxiety Decreased quality of life Depression Anger Negative life changes
Social isolation and loneliness	Unable to do normal activities (community, church, family, and friends) Fewer or no invitations to social events with friends and family Feeling as if you have no one to turn to for support Feeling alone
Medicine management	Difficulty in obtaining medicine Unable to pay for medicine Problems in preparing medicine Trouble in taking medicine on time Taking several medicines for other health problems
Health beliefs	How you view your health and wellness (accept, do not accept, positive, or negative) How well you think you can do health activities for yourself (good, ok, or not well) Your view of how well the heart medicine and treatment works (works well, no change, or does not help)
Caring for yourself (self-autonomy)	You want to do everything for yourself and or make health care decisions whether you can or not Family or friends want to do everything for you and or make health care decisions whether you can or not
Social support and community resources	Family or friends who live close by Church members and social group friends Neighbors Local senior center Home health Grocery store or grocery delivery programs nearby Food pantry Pharmacy nearby Public transportation
Managing HF and other health issues	Managing HF and: Diabetes Neurological disorders

^a^HF: heart failure.

^b^RSV: respiratory syncytial virus.

#### Follow-Up Telephone Sessions

**Figure 2:**
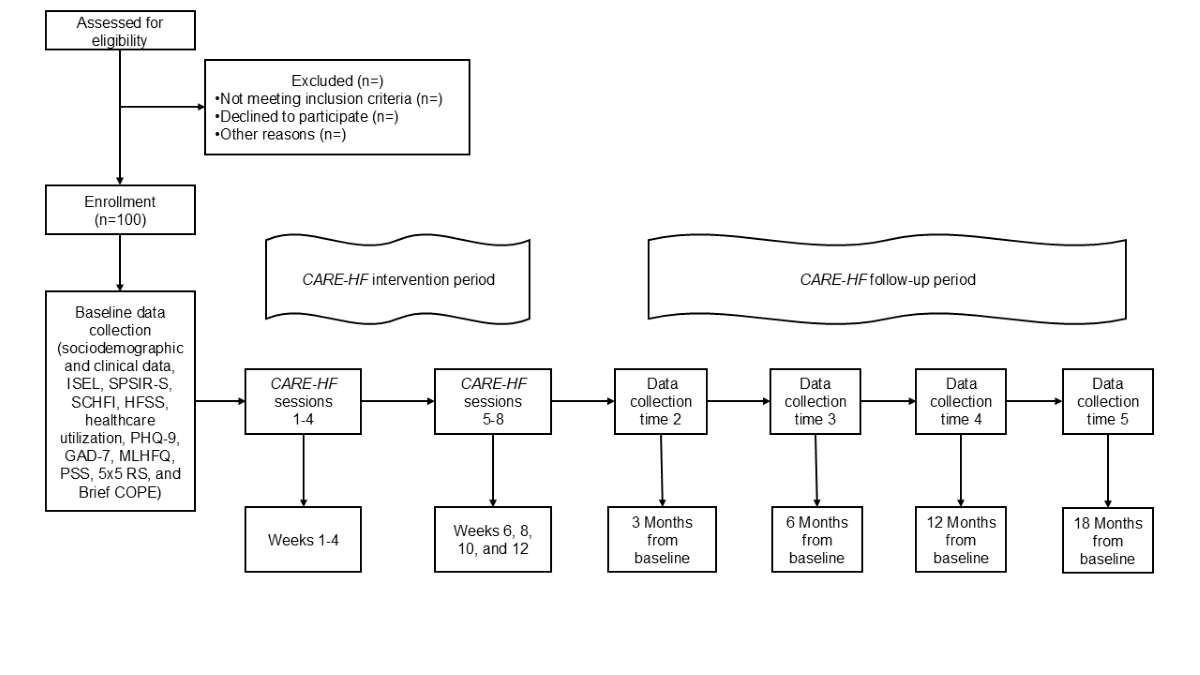
Study overview.

Seven follow-up telephone sessions occur weekly for the first month (sessions 2-4) and then biweekly for the second and third months (sessions 5-8). Each session begins with an evaluation of the previous week’s problem, including the veterans’ perceptions of the effectiveness of the strategies used. If needed, problem-solving guidance is provided or reinforced via verbal feedback from the NI. If the problem improves and no further problem-solving is required, the NI works with the veterans to establish a maintenance plan using rational problem-solving strategies. Veterans then repeat the card sorting task to identify new problems and the iterative five-step, problem-solving process starts over. Both current and new problems are identified by the veterans and examined during each follow-up telephone session. Each follow-up session lasts approximately 30 minutes. Figure 2 provides an overview of the study and associated major activities.

### Intervention Fidelity

Strategies to enhance intervention fidelity are based upon the National Institutes of Health Behavior Change Consortium recommendations [39]. Standardized training for research staff who are conducting inclusion and exclusion screening, data collection, and intervention sessions occurred before study implementation to ensure adequate skill acquisition for the defined roles. The primary investigator trained staff initially until a minimum of 90% accuracy was achieved in covering the key elements of the defined role using role-playing, scenarios, and scripts. Fidelity checks of eligibility screening and informed consent, data collection contacts, and intervention sessions are also performed by the primary investigator every three months using a checklist of key elements for the specific activity to ensure that 90% of core elements are being consistently performed. Retraining of research staff occurs as needed to ensure that they meet the 90% criterion.

### Data Analysis

#### Preliminary Analyses

Descriptive statistics will be computed on all time-invariant and time-varying study variables. Cronbach α values will be calculated for all measures containing multiple items to assess internal consistency. In the case that internal consistency is low (α<0.6), an if-item-deleted analysis will be performed, and items that are not a good indicator for the underlying construct will be excluded.

#### Main Analyses

Multilevel growth curve modeling will be used for the testing of aim 2 or the evaluation of the effectiveness of CARE-HF. For each outcome variable, a series of models will be estimated and compared using incremental fit indices, including the Akaike information criteria and the Bayesian information criteria. The first model is an intercept-only model. Then, a growth curve model with a linear growth component will be estimated. Finally, a model with a quadratic growth component will be tested. The multilevel modeling and restricted maximum likelihood estimation method will be used in R (R Foundation for Statistical Computing) [40]. This method can deal with dropouts and missing data without excluding incomplete cases. In addition to fixed effects, random effects will be estimated.

#### Covariates

Several covariates will be considered for inclusion: age, biological sex, race and ethnicity, and educational attainment. Only covariates that are statistically significant predictors of at least one outcome will be included. That is, the same covariates will be used in all analyses for the primary aim.

### Expected Outcome

We anticipate that the CARE-HF intervention will improve problem-solving, HF self-care, health-related quality of life, resilience, and coping and decrease HF symptoms, health care utilization, depressive symptoms, anxiety, and stress across all time points from baseline.

## Results

Funding for this study was received in October 2022, and recruitment started in April 2023. As of December 2024, a total of 56 veterans have been enrolled, 4 veterans have completed the study, and the remaining veterans are in varying phases of intervention or follow-up. Recruitment is anticipated to end in June 2025, with follow-up data collection continuing until all enrolled veterans have completed the 18-month follow-up data collection period. Data analysis is forthcoming and will be conducted after the study completion.

## Discussion

### Significance of This Study

In recognition of the extraordinary challenges that rural patients encounter in maintaining optimal health, the American Heart Association [22] recently recommended the use of telehealth to increase health equity and provide vital access to cardiovascular supportive care in this population. Although mobile apps and virtual platforms are popular telehealth modalities for intervention delivery, their use in rural populations is plagued by technological challenges [22]. However, the use of telephones to deliver support and problem-solving interventions to rural patients with HF eliminates these technological issues and has been effective in optimizing HF self-care [17,18] and reducing depressive symptoms [41].

With an increasing focus on innovative methods to support physical and mental health and enhance health equity among rural veterans with HF, this study will provide important evidence on the effectiveness of an innovative, telephone-based, support and problem-solving intervention on HF self-care, HF symptoms, health care utilization, depressive symptoms, anxiety, health-related quality of life, stress, resilience, and coping. The CARE-HF intervention has the potential to improve the clinical care of rural veterans with HF, as well as be expanded to other populations. If this novel intervention is found to be effective and sustainable, it can be further tested in a larger randomized clinical trial, with the long-term goal of translating this intervention into clinical care for veterans with HF to enhance HF disease self-management and mental health.

### Limitations

This study has some limitations. First, enrollment is restricted to rural veterans with HF; therefore, generalization of findings may be limited. Second, this is a single-group study that lacks a comparison group, reducing our ability to make meaningful comparisons across outcomes. Third, a lengthy follow-up period is included to examine the sustainability of the intervention effect and assess the need for booster sessions, but this may also contribute to a higher-than-desired attrition rate. Despite these limitations, this study will provide valuable insights for program evaluation and improvement, as well as intervention adherence and sustainability of effect to inform future research.

### Conclusions

This study adapts to the rural sociocultural context and tests a tailored, telephone-based, support and problem-solving intervention designed to help rural veterans manage HF-related problems experienced in the home. If the intervention is effective, it will provide support for a highly accessible, low-cost method to aid rural veterans in maintaining optimal physical and mental health. This study fills an important research gap but, more importantly, provides the basis for future work to evaluate potentially effective and accessible services for rural veterans living with HF.

## Appendix

Multimedia Appendix 1 SPIRIT Checklist.

## Abbreviations

ADAPT-ITT: Assessment, Decision, Adaptation, Production, Topical Experts, Integration, Training, and Testing
CARE-HF: Supporting Physical and Mental Health in Rural Veterans With Heart Failure
COPE-HF: Coping in Heart Failure
HF: heart failure
HIPAA: Health Insurance Portability and Accountability Act
NI: nurse interventionist

## Data Availability

The datasets generated or analyzed during this study are available from the corresponding author on reasonable request.
